# Insights into the diversity of exoplanet atmospheres in the era of JWST

**DOI:** 10.1007/s10509-026-04624-x

**Published:** 2026-08-24

**Authors:** Michael Radica

**Affiliations:** Department of Astronomy & Astrophysics, University of Chicago, 5640 S Ellis Ave., Chicago, 60637 IL USA

**Keywords:** Planets and satellites: atmospheres, Planets and satellites: gaseous planets, Planets and satellites: terrestrial planets, Astronomical data analysis

## Abstract

From religion to science, the quest to understand our place in the universe is one of the fundamental drivers of human progress. Since the discovery of the first exoplanet around a main sequence star in the mid-1990s, our current count of exoplanetary systems has exploded to several thousands. We have even performed in-depth studies of the atmospheres of many distant worlds, yielding insights into the previously unfathomable diversity of planets that exist in the galaxy. In this review article, I summarize work in which I developed several state-of-the-art analysis tools, and applied them to some of the first ever observations of transiting exoplanet atmospheres with the James Webb Space Telescope (JWST). These works provide clear a demonstration of JWST’s revolutionary capabilities: enabling unprecedented insights into the chemical inventory of the atmosphere of a hot-Saturn and hot-Neptune, as well as strict limits on potential atmosphere scenarios of a rocky Venus analogue. These works also encourage new standards for exoplanet data analysis and have laid the groundwork for population-level analyses of atmosphere demographics.

## Introduction

Atmospheric spectroscopy of exoplanets provides a unique window into the physical and chemical processes that govern a planet’s origins, evolution, and potentially even their fate. As we seek to better understand the diverse population of exoplanets — from hot-Jupiters to rocky, potential Earth-twins — we also gain insights into our own solar system. Ultimately, a key goal of exoplanet science is to place our own solar system in the broader context of all known planets in the galaxy.

Since the discovery of the first transiting exoplanet over two decades ago (Charbonneau et al. 2000), the field has made major advances, moving from the detection of planets to in-depth studies of their atmospheres. Particularly for atmosphere studies, space-based observatories like the Hubble (HST) and Spitzer Space Telescopes have played a key role (e.g., Pont et al. 2009; Stevenson et al. 2010; Berta et al. 2012; Kreidberg et al. 2015; Spake et al. 2018; Wakeford et al. 2018). However, although fundamental in their own right, atmosphere studies with HST and *Spitzer* were limited by their precision and wavelength coverage, which in many cases prevented thorough assessments of the chemical inventories of exoplanet atmospheres.

Since its launch in December 2021, JWST has truly lived up to its promise to revolutionize the manner in which we study the atmospheres of transiting exoplanets. With its wide wavelength coverage (∼0.6–12 μm across the instruments most commonly used for exoplanet observations, compared to ∼0.4–1.6 μm for HST), and impeccable spectrophotometric precision (compare spectroscopic errors of ∼400 ppm from HST/WFC3 and <80 ppm for NIRISS/SOSS in 0.025 μm bins for WASP-96 b), JWST has enabled unprecedented insights into the atmospheres of hot giant planets (e.g., JWST Transiting Exoplanet Community Early Release Science Team et al. 2022; Feinstein et al. 2023; Alderson et al. 2023; Rustamkulov et al. 2023; Bell et al. 2024), and enabled the first in-depth studies of smaller and colder worlds like temperate sub-Neptunes (e.g., Madhusudhan et al. 2023; Benneke et al. 2024; Holmberg and Madhusudhan 2024; Schmidt et al. 2025) and rocky planets (e.g., Greene et al. 2023; Lim et al. 2023; Zieba et al. 2023; Moran et al. 2023).

In this review, I present an overview of some of the first exoplanet atmosphere studies with JWST. In Sect. 2, I outline pre-launch, preparatory work on the development of modelling and analysis tools for JWST time series observations (TSOs) of transiting exoplanets. I then apply these tools to early JWST observations of a benchmark hot-Saturn and rocky, Venus analogue in Sect. 3. In Sect. 4, I use the combined powers of JWST and HST to shed new light on a benchmark planet in the hot-Neptune desert — one of the most unique and puzzling demographic features of the exoplanet population. I then conclude and outline some potential future directions for the field in Sect. 5.

## The development of analysis tools for JWST exoplanet atmosphere observations

New observatories will always present users with new challenges; no two observatories, or even detectors, are exactly alike. Although, in some cases, data analysis techniques developed for previous observatories may be largely applicable to new ones, in others routines and analysis pipelines must be developed entirely from scratch; this section outlines key contributions in this area.

### APPLESOSS: a producer of ProfiLEs for SOSS. Application to the NIRISS SOSS mode

Spectral extraction, i.e., the processes of converting 2D detector images into 1D spectra, is one of the most important aspects of data analysis. Ultimately, the 1D spectra are the key observable from any exoplanet dataset. Two commonly employed methods are simple aperture extraction, in which one sums up the flux within a given aperture, and optimal extraction, where the summation is weighted by the instrument point spread function (PSF) to increase signal-to-noise and better reject outliers (Horne 1986). However, both algorithms implicitly assume that the target spectrum is isolated on the detector.

This assumption is axiomatically not valid in the case of the Single Object Slitless Spectroscopy (SOSS; Albert et al. 2023) mode of JWST’s Near Infrared Imager and Slitless Spectrograph (NIRISS; Doyon et al. 2023). SOSS is one of JWST’s major workhorse modes for exoplanet TSOs providing wavelength coverage from 0.6–2.85 μm at a resolution of R∼700. However, since SOSS is a cross-dispersed mode, multiple spectral orders fall onto the detector, two of which partially overlap (e.g., Fig. 3) potentially resulting in strong self-contamination if extracted with usual aperture or optimal extraction routines.

**Fig. 1:**
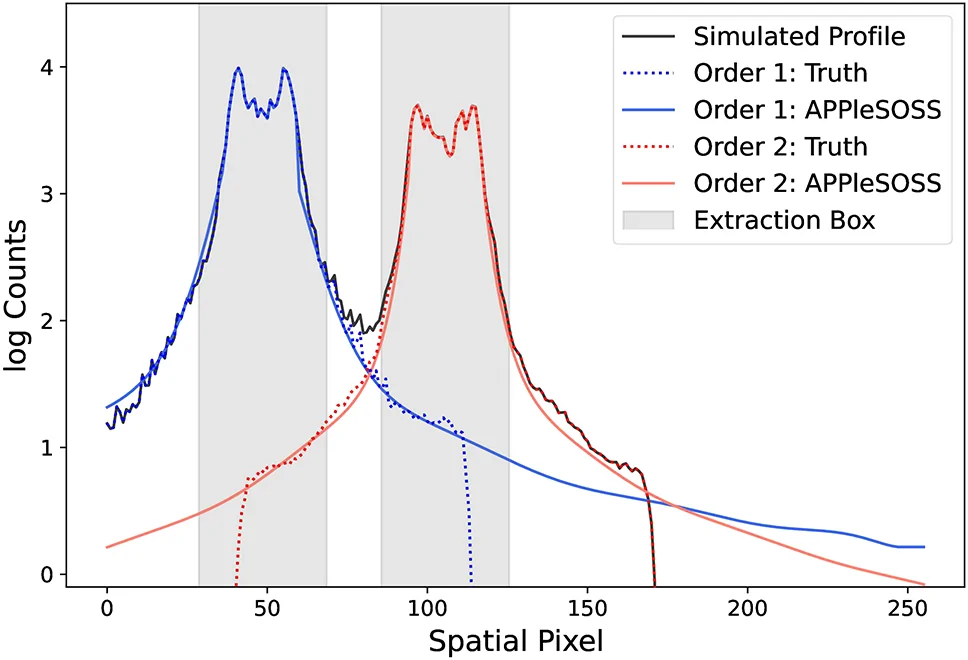
Visualization of the performance of APPLESOSS. In black is the spatial profile of detector column 1000 (∼2 μm in order 1 and ∼0.9 μm in order 2). The dotted blue and red profiles are the “true” profiles for the first and second order respectively which were seeded in the simulation. The solid blue and red lines show the APPLESOSS reconstructions (after scaling to the native counts level). The faded gray boxes indicate the PSF cores. Note that the spatial profiles used as input to the simulation were artificially truncated after ∼100 pixels. Figure from Radica et al. (2022)

The ATOCA (Algorithm to Treat Order ContAmination; Darveau-Bernier et al. 2022) algorithm was developed explicitly to “decontaminate” the SOSS detector, isolating the contributions of the overlapping diffraction orders to a given pixel, and thereby enabling standard extraction techniques to be applied free of self-contamination. However, a key input for this algorithm is the 2D, wavelength-dependent PSF of the SOSS mode. Though theoretical models for this existed even pre-launch, they were shown to not be accurate representations of the SOSS on-sky PSF (Holmberg and Madhusudhan 2023). I, thus, developed a method to empirically estimate the PSF of a given SOSS observation directly from the data. The validation of this algorithm, called APPLESOSS (A Producer of ProfiLEs for SOSS), is shown in Fig. 1.

**Fig. 2:**
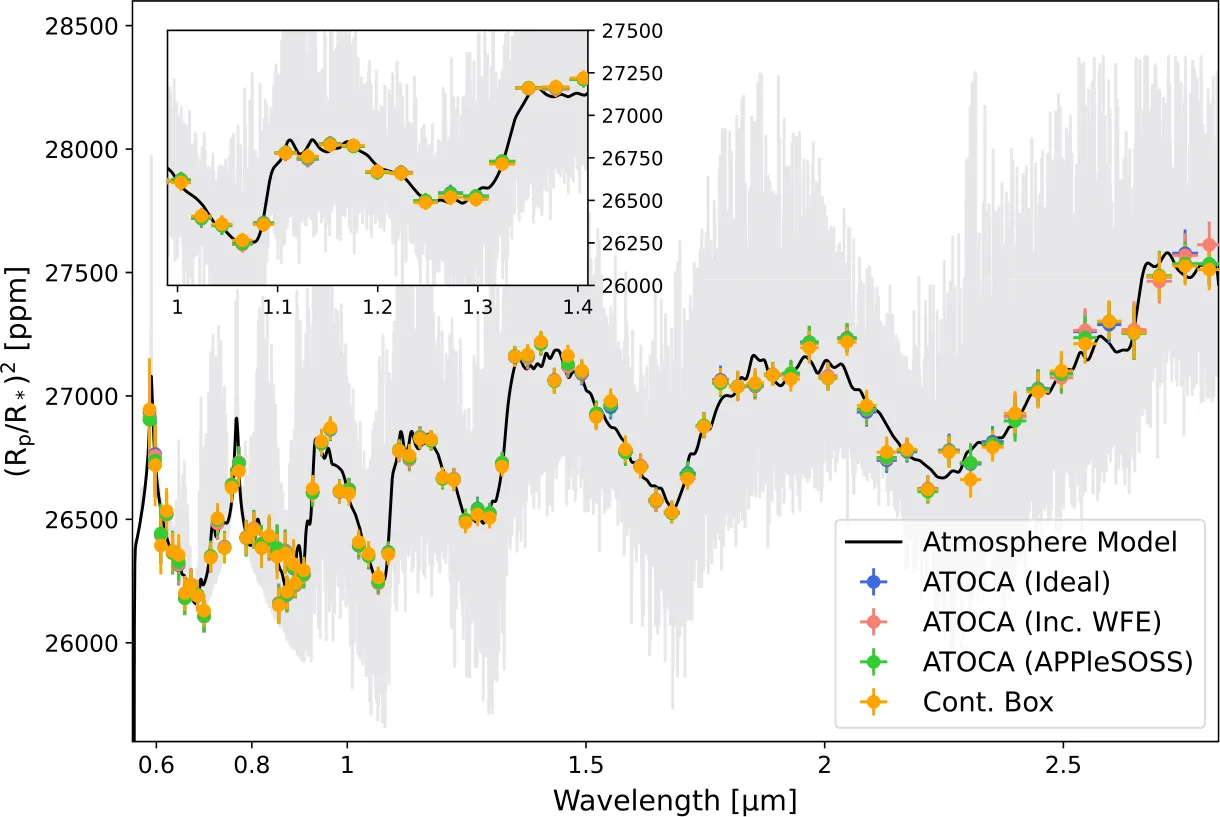
Visualization of the effects of SOSS order self-contamination on simulated data. The coloured points represent transmission spectra derived using several different assumptions for modelling the order self-contamination: an ATOCA extraction using the exact same spectral profile for the extraction and simulation of the underlying data (blue); ATOCA using different wavefront error realizations (Perrin et al. 2014) for the extraction and data simulation profiles (red); ATOCA using an APPLESOSS empirical profile for the extraction (green); and a simple box aperture extraction ignoring the order contamination (orange). In all cases, the transmission spectra agree well within their error bars and with the input atmosphere model shown in black (faded grey at high resolution). Figure from Radica et al. (2022)

However, in both Darveau-Bernier et al. (2022) and Radica et al. (2022) we also demonstrated that for *relative measurements*, e.g., exoplanet transit spectra, the order self-contamination is negligible in the vast majority of cases (e.g., Fig. 2). The self-contamination bias, though, remains extreme for absolute measurements, i.e., of stellar spectra themselves.

### exoTEDRF: an EXOplanet transit and eclipse data reduction framework

Particularly in the JWST era, the ability to obtain robust and reliable atmosphere spectra from raw observations is of paramount importance. For previous-generation observatories like HST and *Spitzer* analysis “pipelines”, which would allow the user to reduce uncalibrated data products through a series of steps with tunable parameters, existed. However, they were often proprietary, meaning that the reproduction of previous studies was difficult, and the bar to entry for newcomers was high.

**Fig. 3:**
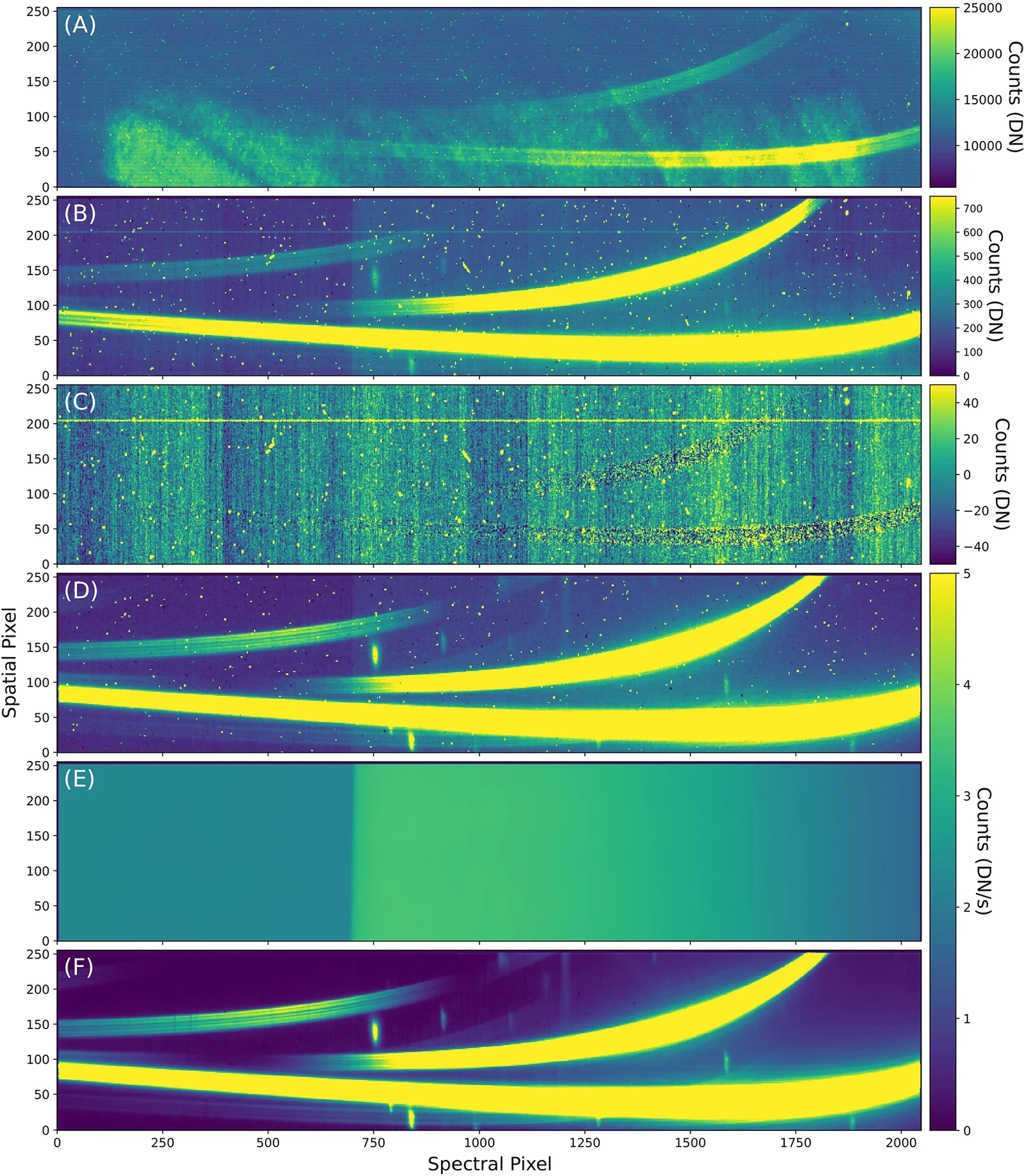
Visualization of the exoTEDRF data products at several stages of the reduction process. (a) A raw, uncalibrated data frame in data numbers (DN). (b): After superbias subtraction and reference pixel correction. (c) Frame (b) after the first background subtraction, and subtraction of the scaled median of all integrations to reveal the 1/f noise (i.e., “flicker” noise; seen here as vertical striping caused by time-varying reference voltage levels during readout). (d) Data frame after ramp fitting and flat field correction. (e): Background model scaled to the flux level of (d). (f): Final calibrated data product. The horizontal stripe near row 200 in panels (b) and (c) is a known artifact resulting from FULL frame resets which occur before each subarray exposure. Figure from Radica et al. (2023)

In order to help address these issues, in Radica (2024) I presented the exoTEDRF analysis pipeline for JWST observations, which has now become one of the most widely used tools in the community. exoTEDRF is an end-to-end analysis pipeline with support for three of JWST’s four instruments: NIRISS (Feinstein et al. 2023), NIRSpec (Ahrer et al. 2025; Radica et al. 2026), and MIRI (Luque et al. 2025). Figure 3 visually summarizes key stages of the exoTEDRF workflow for a NIRISS/SOSS observation. exoTEDRF is entirely open source and features easy-to-follow tutorials — fostering reproducible science and lowering the bar to entry for those working with JWST data for the first time.

## Benchmark planets for the JWST era

One of the ultimate goals of exoplanet science is to understand planets at a *population level*. It is only via these large, demographic studies that we will truly be able to understand the physical processes that e.g., drive planet formation or govern atmosphere evolution. However, it is first necessary to conduct in-depth studies of individual planets in order to calibrate our instruments and determine the correct scientific questions to be asking in the first place.

It is for this reason that establishing benchmark planets, particularly early in the lifetime of any new mission, is particularly valuable. In this section, I outline efforts to establish two such targets for the JWST era at opposite ends of the exoplanet mass continuum: a giant hot-Saturn planet, and a rocky Venus-analogue.

### Awesome SOSS: transmission spectroscopy of WASP-96 b with NIRISS/SOSS

In order to benchmark the capabilities of JWST, one transit of the hot-Saturn WASP-96 b was observed with NIRISS/SOSS as part of the JWST Early Release Observations program. This was one of the very first science observations taken with JWST, and as such provided a perfect opportunity to showcase the revolutionary powers of JWST and benchmark its capabilities on a well-studied target from the HST era.

WASP-96 b (Hellier et al. 2014) is a highly inflated hot-Saturn exoplanet. With a mass of 0.48 $\mathrm{M_{Jup}}$ but a radius of 1.2 $\mathrm{R_{Jup}}$, its atmosphere is incredibly puffy, making it an excellent target for atmosphere observations in transit. WASP-96 b has been targeted with a plethora of observations pre-JWST, including with HST (Nikolov et al. 2022; Yip et al. 2021) and *Spitzer* (Nikolov et al. 2022) from space and the VLT (Nikolov et al. 2018) and Magellan (McGruder et al. 2022) from the ground. These observations indicated a planet with an atmospheric heavy element enrichment roughly equal to that of its host star, and without clouds — the latter finding being particularly unique given the ubiquity of clouds both in the solar system and the broader exoplanet population (e.g., Gao et al. 2021; Samra et al. 2023).

**Fig. 4:**
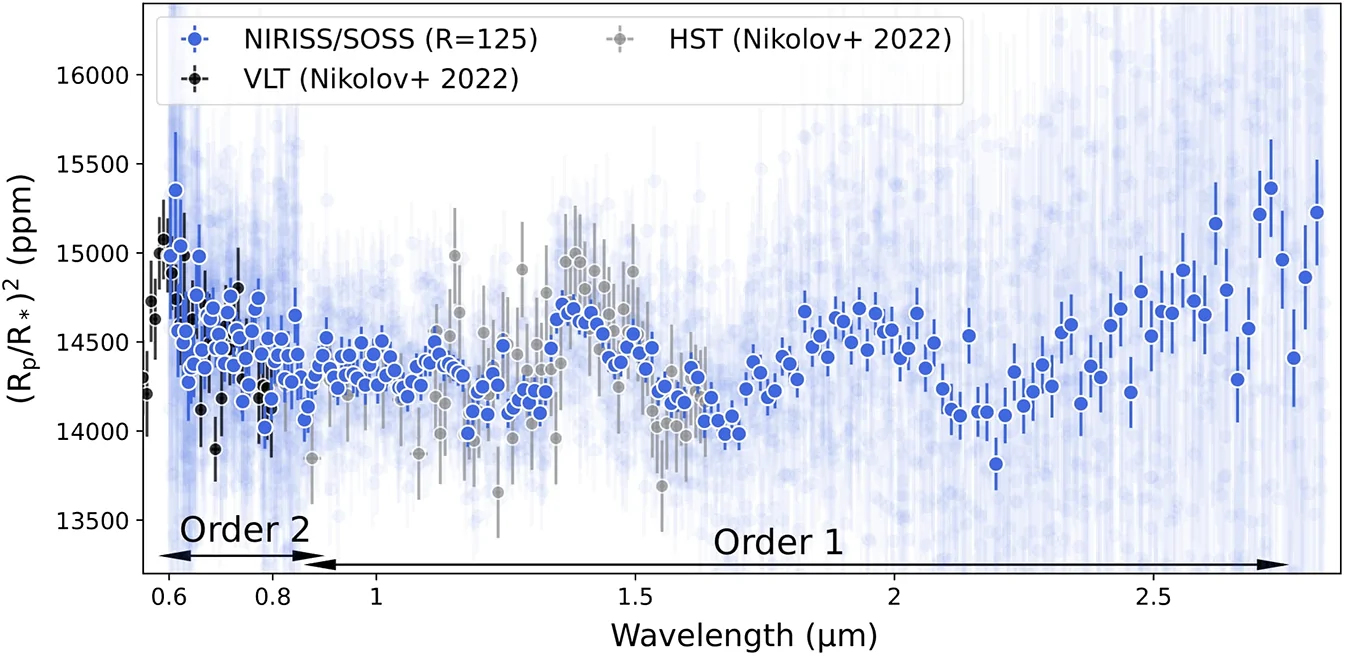
NIRISS/SOSS transmission spectrum of WASP-96 b at pixel resolution (i.e., one transit depth per detector column; faded blue points) and binned to a resolution of R∼125 (blue points). Also shown for comparison are transmission spectra from the VLT/FORS2 (black points) and HST/WFC3 G102 and G141 (grey points), from Nikolov et al. (2018) and Nikolov et al. (2022), respectively. Offsets of 400 and 200 ppm have been applied to the VLT and HST data. Figure from Radica et al. (2023)

Figure 4 compares the transmission spectrum of WASP-96 b from JWST NIRISS/SOSS to previous measurements with HST and the VLT. With one single transit observation, JWST provides higher precision, wider wavelength coverage, and higher spectral resolution than a combination of four transits with HST and VLT. Using both forward and inverse atmospheric modelling techniques, in Radica et al. (2023) and its companion paper Taylor et al. (2023) we obtain a strong detection of H_2_O in the atmosphere of WASP-96 b, weaker detections of Na and K, and marginal evidence for the presence of CO_2_ and CO — both of which were inaccessible to any previous-generation instrument. These chemical species paint a picture of an atmosphere roughly in chemical equilibrium and with a composition broadly matching that of its host star.

We also find evidence for an optical slope in the transit spectrum, which was not seen by any previous observations. Although such a slope could potentially be masked by uncertainties in absolute transit depths when combining observations different instruments (e.g., Yip et al. 2021). Taylor et al. (2023) find that either a highly-broadened Na feature (which is not fully resolved by SOSS) or scattering aerosols could cause the optical slope.

In all, this work provided an exquisite example of the revolutionary capabilities of JWST — paving the way to turn JWST’s powerful gaze to smaller and more difficult to observe planets.

### Promise and peril: stellar contamination and strict limits on the atmosphere composition of TRAPPIST-1 c from JWST NIRISS transmission spectra

The quest to identify an atmosphere around a rocky planet is one of the key goals of exoplanet science. However, to date no firm detection has been made, although tentative evidence for atmospheres have been claimed on several potentially rocky planets (e.g., Hu et al. 2024; Banerjee et al. 2024; Teske et al. 2025; Bello-Arufe et al. 2025; Cherubim et al. 2026). Most searches focus on planets around M-dwarf stars since their small sizes (∼one-tenth to half that of the Sun) amplify the signal of a planet transit. Their cooler temperatures also move their habitable zones (the region around a star where liquid water can potentially exist) to shorter orbital periods where planets are more likely to transit (TRAPPIST-1 JWST Community Initiative et al. 2024).

However, M dwarfs are also considerably more active, have higher XUV outputs, and have longer pre-main sequence phases than earlier-type stars, raising the question of whether a rocky planet orbiting an M dwarf can retain its atmosphere in the first place (TRAPPIST-1 JWST Community Initiative et al. 2024; Pass et al. 2025). Nevertheless, atmosphere searches have continued, targeting in particular the seven Earth-sized planets in the TRAPPIST-1 system. TRAPPIST-1 is an ultra-cool M dwarf with a mass less than a tenth of that of the Sun (Gillon et al. 2016). It hosts seven temperate transiting planets, all roughly the size of the Earth, and with temperatures spanning ∼170–400 K.

We targeted the second-innermost planet in the system, TRAPPIST-1 c with two transits using JWST NIRISS/SOSS in order to attempt to constrain the presence of an atmosphere. Previous observations with HST had ruled out thick, cloud-free, H_2_/He-dominated primary atmospheres (De Wit et al. 2016), and JWST MIRI eclipse observations had also indicated that a thin, CO_2_-dominated atmosphere was unlikely (Zieba et al. 2023). However, there are many potential atmosphere scenarios to which these previous studies were not sensitive (e.g., Lincowski et al. 2023).

We find, though, that both of our visits are highly-contaminated by stellar activity, in the form both of flares and unocculted heterogeneities (i.e., spots, faculae). Unocculted heterogeneities, in particular, can give rise to slopes and even spoof molecular features in transmission spectra, a phenomenon termed the transit light source effect (TLSE; e.g., Rackham et al. 2018, 2019). This had been noted in previous JWST transit observations of rocky planets around M dwarfs (e.g., Moran et al. 2023; May et al. 2023) and of TRAPPIST-1 in particular (Lim et al. 2023). Despite stellar contamination being completely stochastic — the particular spot/facula distribution at any one time is not predictive of later times — we find that both visits serendipitously display the same TLSE realization and the same underlying heterogeneity distribution.

**Fig. 5:**
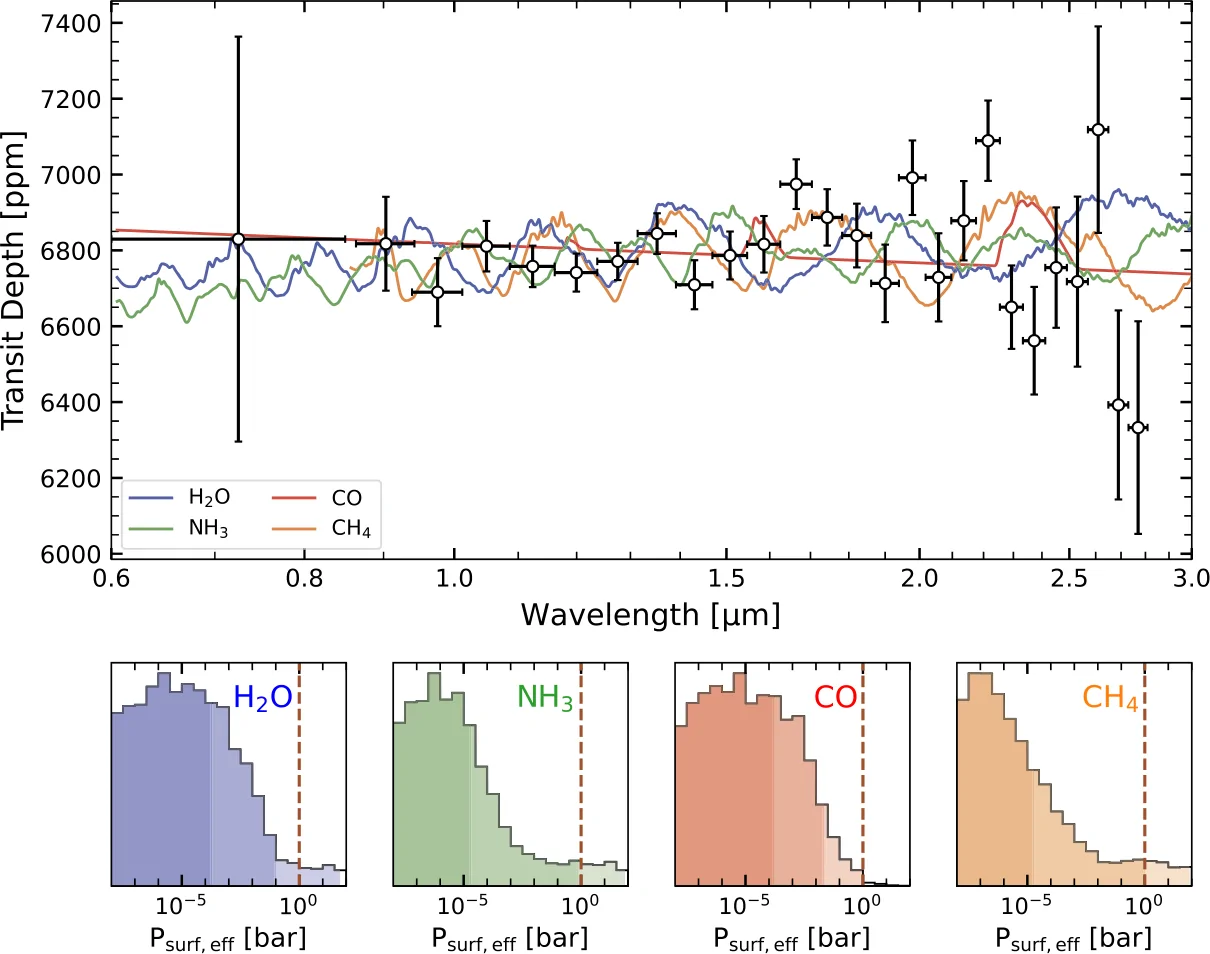
Constraints on high-MMW atmosphere compositions for TRAPPIST-1 c. *Top*: co-added spectrum of TRAPPIST-1 c from the two JWST NIRISS visits, corrected by the best-fitting stellar contamination model (black points) and atmosphere models for deep, 100-bar atmospheres composed of 100% H_2_O (blue), NH_3_ (green), CO (red), and CH_4_ (orange). *Bottom*: marginalized posterior distributions for the effective surface pressure of atmospheres with different compositions. The shaded areas correspond to the 1-σ, 2-σ, and 3-σ upper limits in each scenario. When marginalizing over the impact of stellar contamination, the transmission spectrum disfavours thick, >1-bar atmospheres for all molecules at the 2-σ level. Figure from Radica et al. (2025a)

When marginalizing over the contributions of the TLSE, we do not find any clear evidence for spectral features in the dataset. In particular, the transit spectra can rule out hydrogen-dominated, <300× solar metallicity atmospheres with effective surface pressures down to 10 mbar at the 3-σ level. When considering high-mean-molecular-weight (MMW) or secondary atmospheres, we find that the data disfavours pure H_2_O, NH_3_, CH_4_, or CO atmospheres with pressures of more than 1 mbar at the 2-σ level (e.g., Fig. 5). In all, this work placed some of the most stringent constraints on the presence of an atmosphere on a rocky M dwarf planet, while highlighting the pernicious and stochastic nature of stellar contamination.

## Clouds and survival in the hot-Neptune desert

One of the most remarkable demographic features of the exoplanet population is the hot-Neptune desert — a dearth of planets with masses similar to that of Neptune on orbits of ≲3 d (e.g., Szabó and Kiss 2011; Mazeh et al. 2016; Castro-González et al. 2024). The underlying physical mechanism leading to this desert is thought to be atmosphere escape, with photoevaporation being a leading candidate (e.g., Owen and Wu 2013; Owen and Lai 2018). However, we now know that the desert is not entirely empty, with several planets have being discovered residing deep within this inhospitable region. Chief among these is LTT 9779 b: a roughly Neptune-sized planet which orbits its host star in only 0.79 d — resulting in a temperature of over 2000 K (Jenkins et al. 2020). Moreover, photometric *Spitzer* emission observations of LTT 9779 b indicated that it has somehow retained a substantial H_2_/He-dominated atmosphere (Dragomir et al. 2020; Crossfield et al. 2020).

The manner in which LTT 9779 b, as well as several similar newly-discovered planets, have managed to retain atmospheres in the hot-Neptune desert is still an open question (e.g., Fernández Fernández et al. 2023; Hallatt and Millholland 2026; Vissapragada and Behmard 2025). But as the benchmark planet for the population, it is critical to obtain the maximal information on LTT 9779 b’s atmosphere if we hope to ever resolve this puzzle.

### Muted features in the JWST NIRISS transmission spectrum of hot Neptune LTT 9779 b

**Fig. 6:**
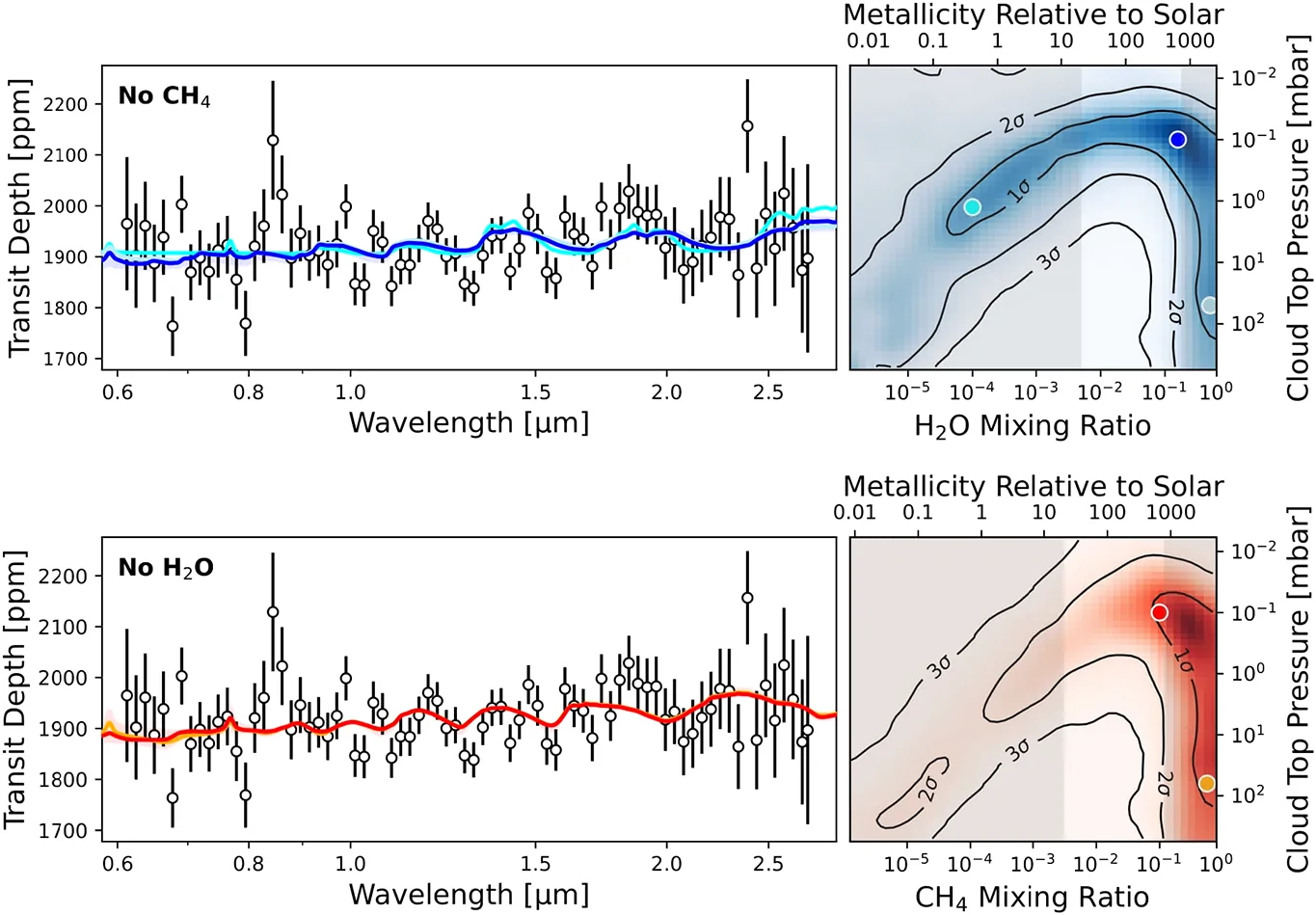
H_2_O- and CH_4_-dominated atmosphere models for LTT 9779 b. *Top left*: the transmission spectrum of LTT 9779 b. Overplotted in dark blue is the best-fitting model from our atmosphere retrievals as well as the 1-σ and 2-σ credible envelopes. Degeneracy between cloud pressure and metallicity allows for solutions with a subsolar metallicity and deeper clouds (light blue), as well as solutions with extremely high (>1000× solar) metallicity and clouds below the observable photosphere (blue-grey). Both data and models are binned to a constant resolution of R = 50 for visual clarity. Note that the blue-grey model is virtually indistinguishable from the dark blue. *Top right*: two-dimensional correlation between the atmosphere metallicity and cloud top pressure in our models. The coloured points mark the parameter space “locations” of the correspondingly coloured atmosphere model. Gray shading denotes regions of parameter space that can be ruled out by population synthesis and interior modelling. *Bottom*: same as the top but for the “No H_2_O”, CH_4_-dominated case. Note that like the dark blue and blue-grey models above, the orange and red models are virtually indistinguishable. Figure from Radica et al. (2024)

We targeted LTT 9779 b with a full-orbit phase curve using JWST NIRISS/SOSS in order to obtain insights into its atmosphere composition and 3D climate. Figure 6 shows the analysis of LTT 9779 b’s NIR transmission spectrum published in Radica et al. (2024). The high-precision transit spectrum rejects a perfectly flat line at >5σ indicating the presence of atmospheric features. However, the amplitudes of these features are highly muted, and as such our models are not able to uniquely identify whether these features are due to H_2_O or CH_4_ — two molecules which have prominant opacity in the NIRISS/SOSS waveband. Our models also indicate a strong degeneracy between the metal enrichment (i.e., metallicity) of the atmosphere and the presence of clouds (right-hand panels in Fig. 6) — which is a well-known degeneracy in transit spectra (e.g., Benneke and Seager 2013; Knutson et al. 2014).

However, when we factor in interior structure modelling, we find that the maximum metal enrichment that LTT 9779 b can have and still respect its observed mass and radius is 850× solar. Moreover, population synthesis analyses by Fortney et al. (2013) showed that Neptune-mass planets do not form with metal enrichments of less than 20× solar. When we include these factors in our analysis, we find that our constraints collapse to prefer solutions with elevated metallicity and clouds high up in the atmosphere. We also find no evidence for atmosphere escape via the metastable He triplet at 1.083 μm, indicating that LTT 9779 b is unlikely to be currently undergoing massive atmosphere loss — a finding that was later confirmed with high-spectral-resolution observations by Vissapragada et al. (2024). A followup analysis of the full-orbit phase curve by Coulombe et al. (2025) also confirmed the presence of clouds in LTT 9779 b’s atmosphere — and in particular asymmetric cloud coverage, with the colder eastern dayside and terminator showing enhanced reflection from clouds which are not present on the warmer western hemisphere.

The finding that LTT 9779 b hosts an atmosphere with high metallicity and enshrouded in reflective clouds led us, in Radica et al. (2024), to suggest a possible feedback loop leading to hot-Neptune atmosphere retention. Since atmosphere escape preferentially removes lighter elements, long periods of escape will naturally lead to an increase in atmosphere metallicity, and in turn to more favourable conditions for cloud formation, even in hot atmospheres (e.g., Wakeford et al. 2017; Hoyer et al. 2023). The formation of highly-reflective clouds can then help shield the planet from the high-energy radiation that drives atmosphere loss, thereby prolonging the atmosphere’s lifetime.

### Constraining the scattered light properties of LTT 9779 b using HST/WFC3 UVIS

The JWST observations discussed in the previous section indicated that LTT 9779 b hosts a cloudy atmosphere, but they do not let us directly assess the composition of these clouds. The SOSS phase curve, as well as CHEOPS optical eclipse observations from Hoyer et al. (2023) indicate a geometric albedo of ∼0.6 — far higher than expected for hot giant planets (e.g., Krenn et al. 2023). Silicate clouds have the required albedo, particularly if their particles are small (Hoyer et al. 2023; Coulombe et al. 2025). An albedo measurement alone, though, is insufficient to directly constrain the composition of cloud condensate particles.

There are two major ways in which the composition of clouds can be probed observationally. The first being the detection of cloud absorption signatures in transit, particularly at mid-infrared wavelengths (e.g., with JWST MIRI; Grant et al. 2023; Inglis et al. 2024; Dyrek et al. 2024). The second entails directly observing scattered light *features* at ultraviolet wavelengths (Sudarsky et al. 2000). Due to the planet’s smaller size compared to the giant planets for which cloud species have been detected with MIRI, LTT 9779 b is a difficult target for this type of observation (Hoyer et al. 2023). We therefore obtained four eclipse observations with the UVIS mode of HST/WFC3 using the G280 grism, which provides spectroscopic wavelength coverage from 0.2–0.8 μm. This was the first ever spectroscopic eclipse observation of an exoplanet with this mode.

**Fig. 7:**
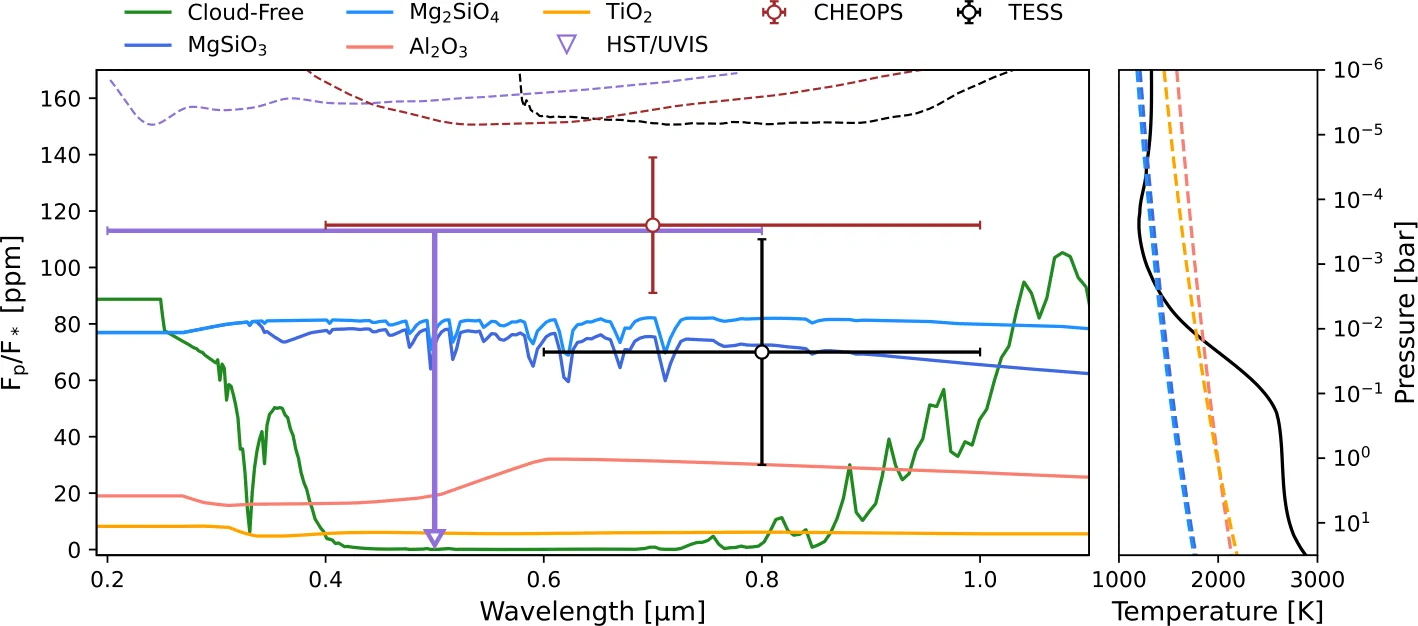
*Left*: comparison of UV/optical photometry with simulated reflectance spectra from the dayside atmosphere of LTT 9779 b. The HST/WFC3 UVIS 3-σ upper limit is denoted by the purple arrow and we include previously published CHEOPS and TESS photometric eclipse depths in brown and black, respectively. The throughput for each instrument is shown with dashed lines at the top of the panel. Overplotted in the solid coloured lines are reflectance spectra generated with VIRGA/PICASO (Batalha et al. 2019) for several plausible cloud condensate species. *Right*: Pressure-temperature (PT) profile for the dayside atmosphere of LTT 9779 b from Coulombe et al. (2025). Condensation curves from Visscher et al. (2010) for all considered species are overplotted in dashed lines. Clouds of a given species can potentially for where the planet PT profile crosses its condensation curve. Figure from Radica et al. (2025b)

Although the goal of the program was to obtain a spectroscopic reflectance spectrum of LTT 9779 b’s atmosphere, and thereby diagnose the particular species of cloud forming in its atmosphere, we did not successfully detect the planet’s eclipse in any of the observations. Figure 7 shows the derived upper limit on the reflected light eclipse depth from the HST/UVIS observations, in context with existing observations from CHEOPS and TESS as well as atmosphere forward model spectra for a variety of cloud condensates. From this forward modelling analysis, we agree with previous studies that silicate clouds like MgSiO_3_ or Mg_2_SiO_4_, particularly if composed of small particles, could provide the necessary albedo. HST/UVIS is also the only observatory-instrument combination able to observe at UV wavelengths until the launch of the eventual Habitable Worlds Observatory, and this study provided an important assessment of its capabilities for Neptune-sized planets.

As the quintessential ultra-hot-Neptune, LTT 9779 b has remained a prominent target for both ground- and space-based observatories which have built upon the above work. In particular, Ashtari et al. (2026) presented a full-orbit phase curve of the planet with JWST’s NIRSpec instrument that observationally confirmed the planet’s high atmospheric metallicity and indicating that its composition is potentially carbon-rich. Vaughan et al. (2026) observed the planet with the Very Large Telescope at high spectral resolution at optical wavelengths and also did not detect the planet’s reflected light spectrum — indicating a potential depletion of the metal oxide TiO in its atmosphere. However, Borsa (2026) noted that it is possible to identify the signal of *stellar* light reflected off of the atmosphere of LTT 9779 b — potentially paving the way for similar future analyses of reflected light from other planets.

## Conclusions & looking forward

The studies on which this review was based leveraged state of the art observatories to obtain unprecedented insights into the atmospheres of benchmark exoplanets, from a hot-Saturn to a rocky Venus analogue. They were some of the very first observations with JWST, and demonstrated its revolutionary precision compared to previous-generation observatories like HST and *Spitzer*. Through them, I also developed robust and widely-used analysis tools for the exoplanet community with the goal of enabling reproducible science and lowering the bar to entry for new scientists.

Although establishing benchmarks is the first step for any new mission, we are now reaching a critical mass of planets with JWST atmosphere observations to allow for studies of entire populations. The work reviewed here has laid the foundations for such studies with JWST. In the near future, space-based (e.g., Ariel; Tinetti et al. 2016) and ground-based observatories (e.g., the European Extremely Large Telescope; Ramsay et al. 2021, Giant Magellan Telescope; Fanson et al. 2020) will also facilitate similar revolutions to JWST and unlock ever deeper insights into the exoplanet population.

## Data Availability

No datasets were generated or analysed during the current study.
